# Different Dynamics for IgG and IgA Memory B Cells in Adolescents following a Meningococcal Serogroup C Tetanus Toxoid Conjugate Booster Vaccination Nine Years after Priming: A Role for Priming Age?

**DOI:** 10.1371/journal.pone.0138665

**Published:** 2015-10-12

**Authors:** Susanne P. Stoof, Anne-Marie Buisman, Debbie M. van Rooijen, Rianne Boonacker, Fiona R. M. van der Klis, Elisabeth A. M. Sanders, Guy A. M. Berbers

**Affiliations:** 1 Centre for Infectious Disease Control, National Institute of Public Health and the Environment (RIVM), Bilthoven, The Netherlands; 2 Department of Immunology and Infectious Diseases, Wilhelmina Children’s Hospital, University Medical Center, Utrecht, The Netherlands; University of Louisville School of Medicine, UNITED STATES

## Abstract

**Background:**

Antibody levels wane rapidly after Meningococcal serogroup C conjugate (MenCC) vaccination in young children, rendering the need for an adolescent booster dose. It is not clear whether circulating memory B cells are associated with persistence of MenC-specific antibody levels.

**Methods:**

Measurement of MenC-specific IgG and IgA memory B cells and levels of serum and salivary MenC-specific IgG and IgA in healthy 10-, 12- and 15-year-olds prior to and one month and one year after a MenCC booster vaccination. All participants had received a primary MenCC vaccination nine years earlier.

**Results:**

The number of circulating MenC-specific IgG memory B cells prior to booster was low and not predictive for MenC-specific IgG responses in serum or saliva post-booster, whereas the number of MenC-specific IgA memory B cells pre-booster positively correlated with MenC-specific IgA levels in saliva post-booster (R = 0.5, P<0.05). The booster induced a clear increase in the number of MenC-specific IgG and IgA memory B cells. The number of MenC-PS-specific IgG memory B cells at 1 month post-booster was highest in the 12-year-olds. The number of MenC-specific memory B cells at one month post-booster showed no correlation with the rate of MenC-specific antibody decay throughout the first year post-booster.

**Conclusions:**

Circulating MenC-specific IgA memory B cells correlate with IgA responses in saliva, whereas circulating MenC-specific IgG memory B cells are not predictive for MenC-specific IgG responses in serum or saliva. Our results are suggestive for age-dependent differences in pre-existing memory against MenC.

## Introduction

*Neisseria meningitidis* is an important cause of septicaemia and meningitis, particularly in young children and adolescents [1]. In industrialized countries, most disease is caused by serogroups B and C [2]. Many countries have introduced a serogroup C polysaccharide-protein conjugate (MenCC) vaccine into their national immunization programme (NIP) [3]. In the Netherlands the MenCC vaccine was implemented into the NIP in 2002 for all children at the age of 14 months. The implementation was accompanied by a catch-up vaccination campaign for children aged between 1–19 years. This catch-up campaign induced large-scale herd protection with low incidence levels of MenC disease up until today [4].

Induction of antigen-specific memory is an important purpose of vaccination. However, a rise in antibody levels after secondary exposure to an antigen takes several days to develop [5–7] while invasion of the bloodstream by meningococci can lead to severe and potentially fatal disease within hours. Therefore, individual protection against invasive meningococcal disease (IMD) is considered to depend mainly on the persistence of sufficient levels of antibody [5, 6].

Previous studies have established an age-dependent difference in persistence of MenC-specific antibody levels following primary MenCC vaccination. In young children (<5 years), persistence is poor and antibody levels decrease to pre-vaccination levels within a few years after primary vaccination [8]. In older children MenC-specific antibody levels can persist for many years after primary MenCC vaccination [9, 10]. This age-dependent difference in persistence has been attributed to maturation of the immune system with age as well as to previous exposure of older individuals to meningococci, rendering the primary vaccination in fact a booster of naturally acquired immunity [11]. In general, several explanations for long-term persistence of increased antibody levels have been postulated. An important one is the generation of long-lived plasma cells that survive and secrete antibody for an extended period of time (>1 year) [12, 13]. Other suggested explanations are chronic exposure of B cells to the antigen through follicular dendritic cells [14] but also repeated stimulation of memory B cells by cross-reactive antigens or through non-specific polyclonal stimulation [7]. Which of these mechanisms underlies the age-dependent difference in antibody persistence after primary MenCC vaccination is still unclear.

To develop an optimal vaccination schedule that establishes long term protection against IMD, it is essential to gain insight into all mechanisms underlying the generation and persistence of MenC-specific antibody levels. Recently we reported the results of MenC-polysaccharide (MenC-PS) specific antibody levels in healthy 10-, 12- and 15-year-olds in response to a MenCC booster vaccination administered nine years after primary MenCC vaccination. Similar to the previously described age-dependent persistence after primary vaccination, we found that persistence of antibody levels in the first year after the booster correlated with age, with best persistence in the oldest age group of our study (15-year-olds) [15]. In addition, we found higher levels of IgA in saliva of the 12- and 15-year-olds in response to the booster compared to the 10-year-olds [16]. These differences between the age groups may be due to the difference in age at primary vaccination, i.e. differences in previously induced MenC-specific memory. Therefore, in a subset of participants from the same study we analyzed the frequency of circulating MenC-PS-specific IgG and IgA memory B cells prior to the booster and kinetics of MenC-PS-specific memory B cells at one month and one year after the booster. In addition, we analyzed the relation between the number of circulating MenC-PS-specific IgG and IgA memory B cells and levels of MenC-PS-specific IgG and IgA in serum and in saliva prior to and throughout the first year after the booster.

## Methods

### Study design and participants

This study was part of a phase IV, single center, open-label study to determine an appropriate age for an adolescent MenCC booster vaccination. Detailed information on the study design and participants has been described previously [15]. Briefly, healthy 10-, 12- and 15-year-old children were recruited in October 2011 from the Utrecht metropolitan area, The Netherlands. Participants had been vaccinated nine years earlier with a single dose of the MenC-polysaccharide conjugated to tetanus toxoid vaccine (MenC-TT, NeisVac C^TM^, Baxter) at 14 months, 2.8 years and 5.8 years, respectively. In addition, all participants were vaccinated according to the Dutch NIP, which includes a tetanus vaccination (DTaP-IPV) at the age of 2, 3, 4 and 11 months and a booster dose (DT-IPV) at the age of 4 years and 9 years. Written informed consent was obtained from both parents and from participants aged ≥12 years. Ethical approval for the study was obtained from a local ethics committee (Verenigde Commissies Mensgebonden Onderzoek). The study was registered at the Dutch Trial register (http://www.trialregister.nl; NTR3521).

### Vaccination and collection of samples

At the beginning of the study, participants received one booster vaccination with the MenC-TT vaccine. Blood samples (5 mL) were taken prior to the booster vaccination (T0) and 1 month (T1) and 1 year (T2) afterwards and stored at -20°C. For the current study, additional blood samples (2x 8 ml) were collected in cell preparation tubes (CPTs) (BD Pharmingen) from a subset of participants. The CPTs were stored overnight (ON) at room temperature. The next day, PBMCs were isolated from the CPTs according to procedures recommended by the manufacturer [17]. Cells were washed with RPMI-1640 medium (Gibco) supplemented with 2% fetal calf serum (FCS, Hyclone), 1% Penicillin and 1% Streptomycin (Lonza). PBMCs were counted by a Coulter Counter (Beckman Coulter, CA, USA) and frozen in a mixture of 80% FCS and 20% DMSO (Sigma-Aldrich) at -80°C ON and subsequently stored at -135°C until use.

### Serological assays

MenC-polysaccharide (MenC-PS) and tetanus toxoid (TT) specific IgG levels were determined using a fluorescent bead-based multiplex immunoassay (MIA) as previously described [15, 18, 19]. Levels of MenC-PS-specific IgA were measured using a modification of the MIA-protocol as previously described [16, 20] with monoclonal mouse-anti-human IgA (Sigma) as primary antibody and R-phycoerythrin-conjugated goat-anti-mouse IgG (Jackson ImmunoResearch) as the subsequent conjugate.

### B cell isolation and stimulation

Stored PBMCs were thawed and B cells extracted by using an anti-CD19 positive selection cocktail (StemCell technologies). Wells of a 96-well round-bottom cell culture plate (Greiner) were filled with 100 μl isolated B cells (5x10^4^ B cells /well). To induce differentiation of memory B cells, 100 μL of polyclonal stimulation medium containing 3 μg/ml CpG (Isogen Life Science) and 10 ng/ml IL-2 (Miltenyi Biotech), IL-10 (BD Pharmingen) and IL-15 (Biosource) was added to each well and incubated for 5 days at 37°C and 5% CO_2_ [17].

### ELISPOT-assays

Multiscreen 96-well plates (Millipore) were coated with 50 μl sterile PBS (Tritium) containing either 5 μg/ml MenC-polysaccharide (NIBSC) conjugated to human serum albumin (NIBSC), 5 μg/ml tetanus toxoid (Baxter), 10 μg/ml goat-anti-human IgG (Cappel) or 10 μg/ml goat-anti-human IgA (Cappel) or solely PBS (blank wells). Coated plates were stored at 4°C until use.

After 5 days of polyclonal stimulation, B cells were harvested, washed, resuspended in AIMV medium (Gibco, Invitrogen) and counted. Wells were blocked with 100 μl AIMV medium for 1 hour before use and filled with 50 μl of cell suspension containing 1x10^5^ B cells. The plates were incubated ON at 37°C and 5% CO_2_. The next day, wells were washed with PBS/0.05% Tween20 (Merck) and goat-anti-human IgG (Sigma Aldrich, 1/5000) or goat anti-human IgA (Sigma 1/2500) was added to bind to secreted IgG or IgA, respectively. After 1 hour of incubation at 37°C, the wells were washed and bound conjugate revealed by adding a substrate (5-bromo-4-chloro-3-indolyl phosphate/Nitro blue tetrazolium, Sigma). Spots were counted using an automatic ELISpot reader (C.T.L. Immunoscan). The number of MenC-PS-specific and TT-specific memory B cells per sample was measured in triplo and the mean number of these three measurements was used for the statistical analyses.

### Statistical analysis

The concentrations of MenC-PS-specific antibody were logarithmically transformed and geometric mean concentrations (GMCs) with 95% confidence intervals (95% CI) were calculated for each age group at each time point. Comparisons between age groups were made using the Mann-Whitney U test. Differences between age groups in proportion of individuals with ≥0.5 detectable memory B cells per 10^5^ B cells at T0 were determined using the χ^2^-test or Fishers exact test. Correlations between the number of memory B cells per 10^5^ B cells and (decay rate of) MenC-PS-specific antibody levels in serum or saliva were made with Spearmans’ rho correlation test. A correlation coefficient (R) between 0–0.40 was considered as a weak correlation, between 0.40–0.70 as moderate and between 0.70–1.00 as strong. All P-values were adjusted for multiple comparisons using the Benjamini and Hochberg False Discovery Rate method. A p-value of <0.05 was considered as statistically significant. Data were analyzed using Excel 2010 software (Microsoft Office) and SPSS statistics 22 (IBM).

## Results

Baseline characteristics of the total study population have been published previously [15]. Table 1 outlines the number of participants with additional blood samples available for the current study together with GMCs of MenC-PS-specific antibody in serum and saliva. GMCs of MenC-PS-specific serum and salivary antibody levels in the current study population were similar to the GMCs in the total study population [15, 16]. Gender distribution was similar across age groups (data not shown). After polyclonal stimulation of B cells, on average 10.5% (±6.3) of the B cell population produced IgG and 3.9% (±1.9) produced IgA. These values were similar throughout the study.

**Table 1:** No. of included samples and MenC-PS-specific IgG and IgA levels in serum and saliva prior to and in response to the MenC conjugate booster vaccination.

			Age at start of study			
	10 years		12 years		15 years	
	MenC-PS-specific		MenC-PS-specific		MenC-PS-specific	
	IgG	IgA	IgG	IgA	IgG	IgA
**Prior to booster (T0)**						
n	17	6	13	10	14	9
Serum antibody; GMC ng/ml (95%CI)	262 (184–374)	14.6 (2.8–75.6)	225 (143–355)	29.0 (12.1–69.2)	478.8 (253.8–903.2)	33.1 (12.3–88.9)
Salivary antibody; GMC ng/ml (95%CI)	0.75 (0.42–1.36)	5.0 (1.9–13.6)	1.37 (0.50–3.74)	10.3 (5.5–19.4)	3.6 (2.1–6.3)	11.7 (7.9–17.2)
**One month post-booster (T1)**						
n	19	7	14	11	18	8
Serum antibody; GMC ng/ml (95%CI)	147,187 (105,345–205,651)	15,830 (4,685–53,492)	256,219 (190,117–345,305)	23,685 (17,108–32,789)	187,106 (125,722–278,459)	38,953 (28,707–52,854)
Salivary antibody; GMC ng/ml (95%CI)	327 (201–534)	72.7 (8.1–653)	709 (347–1,447)	286 (156–523)	1,014 (622–1,652)	226 (110–464)
**One year post-booster (T2)**						
n	19	5	14	11	18	11
Serum antibody; GMC ng/ml (95%CI)	10,281 (6,829–15,476)	813 (257–2,573)	26,859 (19,765–36,499)	2,578 (1,414–4,700)	31,376 (23,384–42,099)	4,329 (2,575–7,276)
Salivary antibody; GMC ng/ml (95%CI)	18.7 (9.0–39.0)	9.2 (1.9–44.2)	38.0 (21.7–66.6)	39.9 (21.3–74.7)	71.8 (43.9–118)	27.2 (13.7–53.8)

**NOTE:** Geometric mean concentrations (GMCs) of serum antibody levels are presented in ng/ml to ease comparison with salivary antibody levels. GMCs of serum and salivary antibody levels of the total study population have been published previously [15,16].

### MenC-PS-specific IgG and IgA memory B cell responses by age

**Fig 1:**
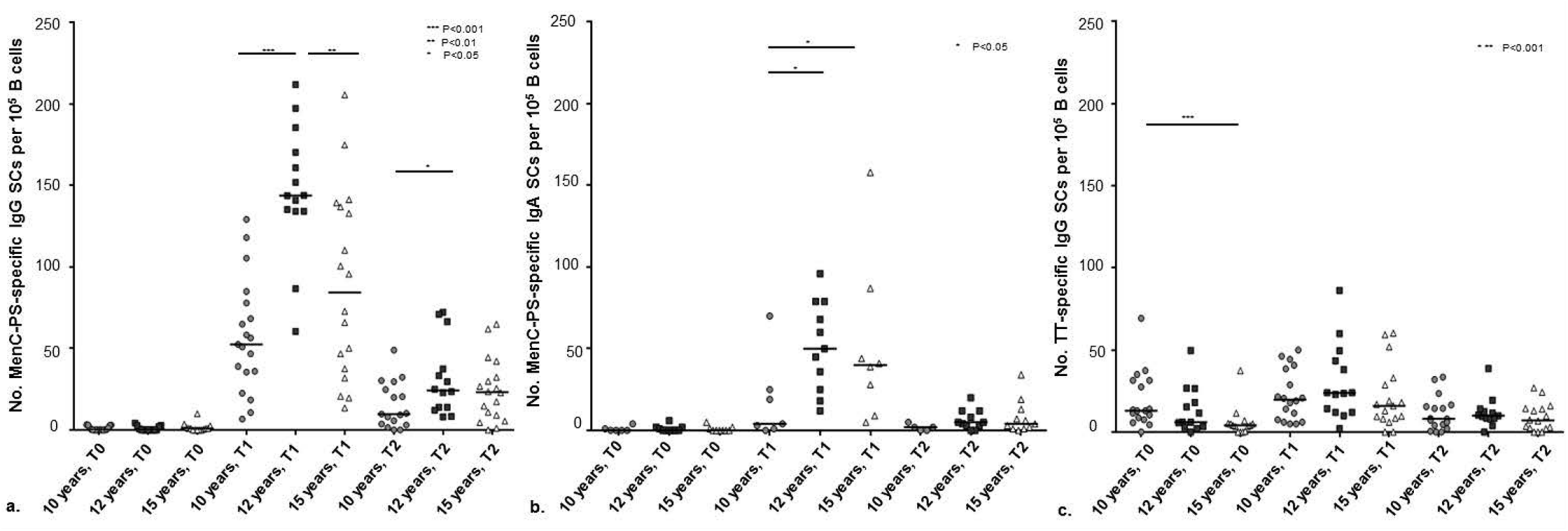
Number of MenC-polysaccharide (MenC-PS) specific IgG memory B cells (a) IgA memory B cells (b) and tetanus toxoid (TT) specific IgG memory B cells (c) per 100.000 B cells in healthy 10-, 12- and 15-year-olds prior to (T0) and one month (T1) and one year (T2) after a booster vaccination with the MenC-PS conjugated tetanus toxoid (MenC-TT) vaccine. All participants had received a primary MenC-TT vaccination 9 years earlier. Memory B cells were measured using ELISPOT-assays. Horizontal bars represent the median number of MenC-PS-specific memory B cells. Comparisons between age groups were made using the Mann-Whitney U test. SC = secreting cells (memory B cells).

Nine years after the single primary MenC-TT vaccination and prior to the MenC-TT booster vaccination, the number of circulating MenC-PS-specific IgG and IgA memory B cells was low and similar across age groups (Fig 1A and 1B). The proportion of participants with ≥0.5 detectable MenC-PS-specific IgG memory B cells per 10^5^ B cells was 35% (6/17) in the 10-year-olds, 38% (5/13) in the 12-year-olds and 57% (8/14) in the 15-year-olds (P = 0.227). The proportion of participants with ≥0.5 detectable MenC-PS-specific IgA memory B cells per 10^5^ B cells was 33% (2/6) in the 10-year-olds, 40% (4/10) in the 12-year-olds and 33% (3/9) in the 15-year-olds (P = 1.000).

The booster induced a clear increase in the number of circulating MenC-PS-specific IgG memory B cells in all age groups with higher numbers in the 12-year-olds at one month post-booster than in the 10- and 15-year-olds (Fig 1A). The booster also induced a clear increase in MenC-PS-specific IgA memory B cells in most of the 12- and 15-year-olds, but only limited in the 10-year-olds (Fig 1B).

One year after the booster, the number of circulating MenC-PS-specific IgG and IgA memory B cells had decreased again in all age groups. The number of MenC-PS-specific IgG memory B cells in the 12-year-olds was still higher than in the 10-year-olds but similar to the 15-year-olds (Fig 1A). No difference was found in the number of MenC-PS-specific IgA memory B cells between age groups at one year post-booster (Fig 1B).

### Correlation between MenC-PS-specific memory B cells prior to booster and MenC-PS-specific antibody levels

Prior to the booster, the number of circulating MenC-PS-specific IgG memory B cells showed no correlation with the pre-booster level of MenC-PS-specific IgG in serum or in saliva. In addition, no correlation was found between the number of circulating IgG memory B cells pre- booster and the level of MenC-PS-specific IgG in serum or in saliva post-booster (Table 2).

**Table 2:** Correlation between number of MenC-PS-specific IgG memory B cells and MenC-PS-specific IgG levels at different time points during the study.

	No. MenC-PS-specific IgGmemory B cells at T0		No. MenC-PS-specific IgGmemory B cells at T1	
	Correlation coefficient (R)	P-value	Correlation coefficient (R)	P-value
**MenC-specific IgG in serum T0**	0.05	0.992	-0.07	0.734
**MenC-specific IgG in serum T1**	-0.05	1.000	**0.53**	**<0.001**
**MenC-specific IgG in serum T2**	-0.08	1.000	**0.38**	**0.012**
**Ratio serum MenC-specific IgG T2/T1**	-0.05	0.922	- 0.02	0.902
**MenC-specific IgG in saliva T0**	0.12	1.000	0.14	0.460
**MenC-specific IgG in saliva T1**	0.07	1.000	**0.42**	**0.005**
**MenC-specific IgG in saliva T2**	-0.09	1.000	0.28	0.075
**Ratio saliva MenC-specific IgG T2/T1**	0.18	1.000	-0.06	0.700

**NOTE**: T0 = prior to MenC-TT booster vaccination, T1 = 1 month after MenC-TT booster vaccination, T2 = 1 year after MenC-TT booster vaccination. No. of memory B cells were measured using ELISPOT. Correlations (R) were analyzed using the Spearman’s rho correlation test. P-values (P) were adjusted for multiple comparisons using the Benjamini and Hochberg False Discovery Rate method. Ratio T2/T1 represents fold decrease (decay) in IgG levels between T1 and T2.

The number of circulating MenC-PS-specific IgA memory B cells prior to the booster showed a strong correlation with the pre-booster level of MenC-PS-specific IgA in serum, but no correlation with the IgA level in saliva. The number of circulating MenC-PS-specific IgA memory B cells prior to the booster showed a moderate correlation with the IgA level in saliva at one month and one year post-booster but no correlation with the level of MenC-PS-specific IgA in serum at these time points (Table 3).

**Table 3:** Correlation between number of MenC-PS-specific IgA memory B cells and MenC-PS-specific IgA levels at different time points during the study.

	No. MenC-PS-specific IgA memory B cells T0		No. MenC-PS-specific IgA memory B cells T1	
	Correlation coefficient (R)	P-value	Correlation coefficient (R)	P-value
**MenC-specific IgA in serum T0**	**0.65**	**<0.001**	0.33	0.154
**MenC-specific IgA in serum T1**	0.33	0.194	0.38	0.112
**MenC-specific IgA in serum T2**	0.20	0.407	**0.50**	**0.036**
**Ratio serum MenC-specific IgA T2/T1**	-0.09	0.726	0.29	0.201
**MenC-specific IgA in saliva T0**	0.13	0.621	0.23	0.303
**MenC-specific IgA in saliva T1**	**0.50**	**0.048**	**0.50**	**0.048**
**MenC-specific IgA in saliva T2**	**0.47**	**0.041**	**0.49**	**0.035**
**Ratio saliva MenC-specific IgA T2/T1**	-0.28	0.266	-0.22	0.289

**NOTE**: T0 = prior to MenC-TT booster vaccination, T1 = 1 month after MenC-TT booster vaccination, T2 = 1 year after MenC-TT booster vaccination. No. of memory B cells were measured using ELISPOT. Correlations (R) were analyzed using the Spearman’s rho correlation test. P-values (P) were adjusted for multiple comparisons using the Benjamini and Hochberg False Discovery Rate method. Ratio T2/T1 represents fold decrease (decay) in IgA levels between T1 and T2.

### Correlation between MenC-PS-specific memory B cells at one month post-booster and persistence of MenC-PS-specific antibody levels in first year post-booster

The number of circulating MenC-PS-specific IgG memory B cells at one month after the booster showed a moderate correlation with MenC-PS-specific IgG levels in serum and in saliva at one month post-booster but a weak correlation with IgG levels in serum at one year post-booster (Table 2). Similar results were found when the number of circulating MenC-PS-specific IgG memory B cells at one month post-booster were related to the level of serum bactericidal antibody (SBA), i.e. functional antibody levels (S1 Table). Previously, we found better persistence of antibody levels in the 15-year-olds after the booster, represented by a lower rate of decay in MenC-PS-specific IgG levels throughout the first year post-booster. Here, we assessed the relation between the number of IgG memory B cells at one month post-booster and the rate of decay in IgG levels throughout the first year post-booster (ratio IgG T2/T1), but found no correlation (Table 2).

The number of circulating MenC-PS-specific IgA memory B cells at one month post-booster correlated moderately with the level of MenC-PS-specific IgA in saliva at one month post-booster, but not with the IgA level in serum. Furthermore, the number of IgA memory B cells at one month post-booster showed a moderate correlation with the level of IgA in both serum and saliva at one year post-booster (Table 3). Similar to MenC-PS-specific IgG, no correlation was found between the number of circulating MenC-PS-specific IgA memory B cells at one month post-booster and the rate of decay in serum or salivary IgA (ratio IgA T2/T1) levels throughout the first year post-booster (Table 3).

### TT-specific IgG memory B cells and TT-specific IgG levels in serum

In line with the last tetanus booster received at the age of 9 years, the number of TT-specific IgG memory B cells prior to the MenC-TT booster in this study was highest in the 10-year-olds and lowest in the 15-year-olds. The number of TT-specific IgG memory cells only slightly increased in response to the MenC-TT booster and decreased again in the subsequent year. No differences were found between the age groups in the number of TT-specific memory B cells at one month or at one year post-booster (Fig 1C).

Throughout the study, the number of circulating TT-specific IgG memory B cells showed a moderate correlation with the level of TT-specific IgG in serum. The number of TT-specific IgG memory B cells at one month post-booster showed no correlation with the rate of decay in TT-specific IgG levels (ratio IgG T2/T1) throughout the first year post-booster (S2 Table).

## Discussion

In the current study, we found low numbers of circulating MenC-PS-specific IgG and IgA memory B cells nine years after a single primary MenC-TT vaccination. The MenC-TT booster induced a clear increase in the number of MenC-PS-specific IgG and IgA memory B cells in all age groups, though for IgA the increase was less pronounced in the 10-year-olds. The number of circulating IgG memory B cells prior to the booster was not predictive of the booster response in serum or salivary MenC-PS-specific IgG. The number of IgA memory B cells prior to the booster correlated with the level of MenC-PS-specific IgA in saliva at one month and one year post-booster. No correlation was found between the number of MenC-PS-specific memory B cells at one month post-booster and the rate of MenC-PS-specific antibody decay in serum or saliva throughout the first year post-booster.

The low number of circulating MenC-PS-specific IgG memory B cells prior to the booster was anticipated since a study from the UK performed six years after priming also showed low numbers of MenC-PS-specific memory B cells [21]. It is assumed that MenC-PS-specific IgG memory B cells only transiently circulate through the blood after vaccination and subsequently reside in peripheral lymphoid tissue [21–23]. Re-exposure to the antigen induces recruitment of these cells. Large-scale catch-up vaccination of young children up to young adults in both the UK (1999) and the Netherlands (2002) induced herd protection due to vaccine-induced eradication of MenC carriage and transmission [4, 24, 25]. To date, MenC carriage levels are still low [26]. In other words, there is currently no natural trigger for recruitment of MenC-PS-specific IgG (and IgA) memory B cells and their frequency in the circulation prior to the booster is therefore understandably low.

The booster induced high MenC-PS-specific IgG levels and a marked increase in the number of MenC-specific IgG memory B cells in all age groups, indicative of a clear memory response. In line with the UK study performed six years after priming, we found that the number of circulating MenC-PS-specific IgG memory B cells prior to the booster was not predictive for the booster response in MenC-PS-specific IgG [21]. In the UK study no difference was found between age groups (ranging from 7- to 12-year-olds) in the number of IgG memory B cells at one month post-booster and the authors concluded that the memory B cell booster response was not dependent on age at primary vaccination [21]. However, the 12-year-olds in our study showed markedly higher numbers of IgG memory B cells at one month post-booster than the 10- and 15-year-olds. The fact that we did find a difference between age groups may be explained by the use of isolated B cells in the current study instead of PBMCs, which increased the sensitivity of the assay [17]. Nevertheless, we have no clear explanation why we specifically found higher numbers of MenC-PS-specific memory B cells in the 12-year-olds at one month post-booster. It could be explained by differences in maturation of B-cells subsets during puberty due to differences in hormone levels. Alternatively, the 12-year-olds may have had a higher number of pre-existing memory B cells [23, 27, 28]. Considering the current absence of MenC circulation, a difference in pre-existing memory must have been either present before or induced by primary MenCC vaccination. Participants were primed at the age of 14 months, ~3 years or ~6 years, respectively. It would have been more logical if the oldest age group (15-year-olds) had developed the highest number of memory B cells in response to the booster, since their immune system was most mature at time of priming and they were more likely to be naturally exposed prior to primary MenCC vaccination. [29]. Perhaps different ages at priming leads to development of qualitatively different memory B cells, e.g. with a difference in ability to differentiate into either memory B cells or (long-lived) plasma cells in response to a booster vaccination. More research is required to establish the role of age at priming on the response to a booster vaccination.

The number of MenC-PS-specific IgG memory B cells at one month after the booster showed a weak correlation with the level of MenC-PS-specific IgG at one year post- booster. This may suggest a direct relation through ongoing differentiation of memory B cells and production of IgG. However, the number of IgG memory B cells at one month post-booster showed no correlation with the rate of antibody decay throughout the first year post-booster. In addition, the number of IgG memory B cells at one month post-booster was not highest in the 15-year-olds which contrasts with best persistence of IgG in the 15-year-olds after the booster [15]. Together, these findings merely suggest that the number of circulating MenC-PS-specific IgG memory B cells found at one month post-booster represents the size of the MenC-specific immune response evoked by the booster. The number of MenC-PS-specific IgG memory B cells is probably proportional to the level of MenC-PS-specific IgG produced by (long-lived) plasma cells, but not directly related to the (persistence of) IgG-levels. A similar suggestion was made by Blanchard-Rohner et al who found a positive correlation between the number of memory B cells at 5 months of age (one month after infant primary series) and antibody levels at 12 months [23]. Increased persistence of MenC-PS-specific IgG with age after the booster in our study may be explained by induction of a higher number of long-lived plasma cells in the older age groups. Whether this is due to difference in age at booster or to difference in age at priming remains to be elucidated.

To our knowledge, this is the first study investigating frequencies of circulating MenC-PS-specific IgA memory B cells upon MenCC vaccination. The number of circulating IgA memory B cells prior to the booster showed a moderate correlation with post-booster levels of IgA in saliva. Furthermore, the number of MenC-PS-specific IgA memory B cells found at one month post-booster correlated with IgA levels in both serum and saliva at one year post-booster. These findings suggest different dynamics for MenC-PS-specific IgA memory B cells compared to MenC-PS-specific IgG memory B cells. We previously reported a correlation between serum and salivary MenC-PS-specific IgA levels following the MenCC booster but also between the level of salivary MenC-PS-specific IgA and secretory component [16]. The latter is suggestive for local production of IgA in saliva. Interestingly, we previously found a higher MenC-PS-specific IgA1/IgA2 ratio post-booster in serum than in saliva which may suggest relatively more IgA2 production at the mucosal site (unpublished data). Taken together, our findings may imply that the parenteral booster vaccination generated a proportional subset of MenC-PS-specific IgA B cells–likely plasma blasts—which homed to the mucosal site and locally produced IgA. A similar correlation between circulating IgA B cells and salivary IgA has previously been obtained following pneumococcal vaccination [30]. This was preceded by natural induction of immunity at the mucosal site due to pneumococcal carriage. If the same is true for salivary IgA in response to meningococcal vaccination, this would imply that a substantial part of our study population had natural immunity induced at the mucosal site prior to primary vaccination. This seems rather unlikely, considering the young age at primary vaccination of our study participants and the previously described low meningococcal carriage levels in young children [29]. Nevertheless, MenC-PS-specific IgA responses in our study were highest in the 12- and 15-year-olds who were older at time of priming and therefore more likely to be exposed than the 10-year-olds. Importantly, our results should be interpreted with caution since the number of samples available for the analysis of MenC-PS-specific IgA memory B cells in our study was low and the results only provide indirect evidence.

Important strengths of the current study are the use of longitudinal samples as well as increased accuracy of the ELISPOT assays by using isolated B cells instead of PBMCs. However, the number of samples available for the current study was rather low, particularly for IgA. Moreover, we studied circulating B cells which may not be fully representative for the majority of memory B cells residing in peripheral lymphoid tissue. In addition, we studied circulating memory B cells at one month and one year post-booster, while the numbers of circulating memory B cells probably peak between day 7 and day 14 upon MenCC booster vaccination [21, 23, 31]. However, the number of circulating MenC-PS-specific IgG memory B cells were still clearly elevated one month after MenC conjugate booster vaccination in previous studies [21, 23, 31], indicating that this time point is still representative for kinetics of circulating memory B cells upon MenCC booster vaccination.

To conclude, nine years after a single primary MenC-TT vaccination the number of circulating MenC-PS-specific IgG and IgA memory B cells was low, but increased significantly in response to the booster vaccination. The number of circulating IgG memory B cells was not predictive for MenC-PS-specific IgG responses in serum or saliva. Increases in IgG memory B cells were most pronounced in the 12-year-olds and may be indicative for differences in pre-existing memory. The higher number of MenC-PS-specific IgA memory B cells in the older age groups and the correlations between IgA memory B cells and salivary IgA levels may suggest differences in natural induced immunity prior to (primary) vaccination. In view of the current low MenC carriage levels and for the purpose of developing an optimal vaccination schedule against invasive meningococcal disease, further research is required to establish the role of age at priming on booster responses and the impact of (absence of) natural induced immunity on the persistence of MenC-PS-specific antibody after primary and booster vaccination.

## Supporting Information

S1 Dataset Minimal data set underlying the findings. (PZF)

S1 Table Correlation between number of MenC-PS-specific IgG and IgA memory B cells and Serum Bactericidal Antibody assay (SBA) titer. (DOCX)

S2 Table Correlation between number of TT-specific IgG memory B cells and TT-specific IgG levels at different time points during the study. (DOCX)
