# Supplementary material for: Different Dynamics for IgG and IgA Memory B Cells in Adolescents following a Meningococcal Serogroup C Tetanus Toxoid Conjugate Booster Vaccination Nine Years after Priming: A Role for Priming Age?
Source: PLoS One. 2015 Oct 12;10(10):e0138665. doi: 10.1371/journal.pone.0138665 (PMC4601787; doi:10.1371/journal.pone.0138665)
Supplement: S1 Table — (DOCX) [file pone.0138665.s002.docx]

**Supplementary Table 1 Correlation between number of MenC-PS-specific IgG and IgA memory B cells and Serum Bactericidal Antibody assay (SBA) titer**

|  | MenC-PS-specific memory B cells T0 | | | | MenC-PS-specific memory B cells T1 | | | |
| --- | --- | --- | --- | --- | --- | --- | --- | --- |
|  | **IgG** | | **IgA** | | **IgG** | | **IgA** | |
|  | **R** | **P** | **R** | **P** | **R** | **P** | **R** | **P** |
| **SBA titer T0** | 0.06 | 0.783 | 0.51 | 0.090 | -0.10 | 0.604 | -0.02 | 0.929 |
| **SBA titer T1** | -0.05 | 0.773 | 0.03 | 1.000 | **0.45** | **0.005** | 0.32 | 0.191 |
| **SBA titer T2** | -0.138 | 0.557 | 0.08 | 0.899 | **0.43** | **0.006** | 0.46 | 0.086 |

**NOTE**: T0=prior to MenCC booster vaccination, T1=1 month after MenC-TT booster vaccination, T2= 1 year after MenC-TT booster vaccination. Correlations (R) were analyzed using the Spearman’s rho correlation test. P-values (P) were adjusted for multiple comparisons using the Benjamini and Hochberg False Discovery Rate method.
