# Supplementary material for: Different Dynamics for IgG and IgA Memory B Cells in Adolescents following a Meningococcal Serogroup C Tetanus Toxoid Conjugate Booster Vaccination Nine Years after Priming: A Role for Priming Age?
Source: PLoS One. 2015 Oct 12;10(10):e0138665. doi: 10.1371/journal.pone.0138665 (PMC4601787; doi:10.1371/journal.pone.0138665)
Supplement: S2 Table — (DOCX) [file pone.0138665.s003.docx]

|  | No. TT-specific IgG ASCs T0 | | No. TT-specific IgG ASCs T1 | |
| --- | --- | --- | --- | --- |
|  | **R** | **P** | **R** | **P** |
| **TT-specific IgG in serum T0** | **0.62** | **<0.001** | 0.17 | 0.254 |
| **TT-specific IgG in serum T1** | **0.46** | **0.004** | **0.60** | **<0.001** |
| **TT-specific IgG in serum T2** | **0.52** | **<0.001** | **0.46** | **0.002** |
| **Ratio TT-specific IgG T2/T1** | 0.09 | 0.630 | -0.21 | 0.190 |

**Supplementary table 2. Correlation between number of TT-specific IgG memory B cells and TT-specific IgG levels at different time points during the study.**

**NOTE**: T0=prior to MenC-TT booster vaccination, T1=1 month after MenC-TT booster vaccination, T2= 1 year after MenC-TT booster vaccination. No. of memory B cells were measured using ELISPOT. Correlations (R) were analyzed using the Spearman’s rho correlation test. P-values (P) were adjusted for multiple comparisons using the Benjamini and Hochberg False Discovery Rate method. Ratio IgG T2/T1 represents the fold decrease (decay) in IgG levels between T1 and T2.
